# Lymphocyte subsets in untreated thalassemia patients: differences by genotype and age

**DOI:** 10.3389/fimmu.2026.1832294

**Published:** 2026-06-03

**Authors:** Renbin Zhao, Xiangmei Yao, Shuai Feng, Peng Hu, Yunlian Zou, Zefeng Yang, Jinping Zhang, Xin Guan, Chaoran Zhang, Zhongyu Wang, Jie Zhang, Zengzheng Li, Yajie Wang

**Affiliations:** 1 Department of Hematology, The First People’s Hospital of Yunnan Province, Affiliated Hospital of Kunming University of Science and Technology, Kunming, China; 2 Yunnan Province Clinical Research Center for Hematologic Disease, The First People’s Hospital of Yunnan Province, Kunming, China; 3 Yunnan Provincial Clinical Medical Center for Blood Diseases and Thrombosis Prevention and Treatment, The First People’s Hospital of Yunnan Province, Kunming, China; 4 Department of Integrated Traditional Chinese and Western Medicine, The First People’s Hospital of Yunnan Province, The Affiliated Hospital of Kunming University of Science and Technology, Kunming, Yunnan, China; 5 School of Medicine, Kunming University of Science and Technology, Kunming, China; 6 Department of Medical Genetics, The First People’s Hospital of Yunnan Province, The Affiliated Hospital of Kunming University of Science and Technology, Kunming, Yunnan, China

**Keywords:** adults, lymphocytes, pediatric, regulatory t cells, thalassemia

## Abstract

**Background:**

The Lymphocyte subsets in treatment-naïve patients and their variations among different genetic subtypes and age groups remain poorly characterized.

**Methods:**

To characterize the distribution of lymphocyte subsets in treatment-naïve thalassemia patients, stratified by both genetic subtype and age group (pediatric vs. adult), compared to healthy controls. This investigation included 535 participants, comprising 307 healthy controls (104 pediatric and 203 adult patients) and 228 untreated thalassemia patients (123 α-thalassemia, 83 β-thalassemia, and 22 compound α+β-thalassemia cases). Genotyping was performed using NGS, while flow cytometry was utilized to quantify peripheral blood lymphocyte subsets.

**Results:**

Pediatric patients: All thalassemia subtypes (α, β, α+β) showed significantly higher Tregs frequencies than healthy pediatric controls (all P < 0.0001). Pediatric α+β-thalassemia patients had lower CD3+ T-cell proportions than β-thalassemia patients.Adult patients: Adult β+-thalassemia patients had significantly higher B-cell and Tregs frequencies than healthy adult controls (P = 0.037, P = 0.034). Adult α+β-thalassemia patients had lower CD4+ T-cell percentages than healthy adult controls (P = 0.040).Age-group comparisons: Tregs levels were significantly higher in healthy adults than in healthy pediatric individuals (P < 0.0001). Pediatric β+-thalassemia patients had lower CD3+ T-cell frequencies than adult β+-thalassemia patients (P = 0.009). Pediatric patients with Hb Constant Spring heterozygosity or Codons 41/42 (-TTCT) β0 heterozygosity also had lower CD3+ and CD4+ T-cell proportions than corresponding adult patients (all P < 0.05).

**Conclusions:**

This study clarified the distribution of lymphocyte subsets in treatment-naïve patients with thalassemia. It was found that the number of Tregs in healthy pediatric was significantly lower than that in healthy adults. Moreover, the level of Tregs was markedly higher in pediatric with thalassemia than in healthy pediatric. These findings may help deepen the understanding of immune dysregulation in thalassemia patients and facilitate the formulation of clinical management strategies.

## Introduction

Thalassemia is a group of inherited hemolytic anemias caused by defects in globin genes that impair hemoglobin synthesis, primarily classified into α-thalassemia and β-thalassemia. The molecular pathology involves various types of genetic deletions or point mutations (1).As one of the most widely distributed monogenic disorders globally, the disease exhibits high prevalence rates in regions extending from the Mediterranean, Middle East, and Africa to Southeast Asia. With population migration, its prevalence continues to rise in Europe and North America, making it a major public health concern worldwide (1, 2). Approximately1.67% of the global population carries thalassemia genes, totaling 240 to 400 million carriers, with 2 to 3 million severe cases (1, 2). The core pathological features include chronic hemolysis, ineffective erythropoiesis, and iron overload, which can lead to multi-system complications such as hepatosplenomegaly, skeletal deformities, and cardiovascular damage over time (1). Immune system dysfunction represents a significant accompanying pathological state. As early as 1979, Musumeci et al. first reported abnormal lymphocyte alterations in splenectomized patients with severe thalassemia (3). Subsequent studies further confirmed that splenectomized adult β-thalassemia major patients showed significantly higher percentages of B cells and T cells compared to healthy individuals, while severely transfused patients exhibited markedly impaired T lymphocyte proliferation and effector functions (4–7). This coexistence of immunodeficiency and chronic inflammation ultimately leads to substantially increased susceptibility to infections, becoming one of the primary causes of mortality (8, 9).

Current investigations into thalassemia and lymphocyte subsets predominantly concentrate on patients receiving clinical interventions, including splenectomy, chronic transfusion therapy, iron chelation, and hematopoietic stem cell transplantation, whereas studies examining the baseline immune status of treatment-naïve patients remain limited. Moreover, existing literature fails to comprehensively characterize lymphocyte subset distributions across distinct genetic subtypes (α-thalassemia, β-thalassemia, and compound α/β-thalassemia) or different age demographics (pediatric versus adult patients). To address these knowledge gaps, this study conducts a systematic analysis of peripheral blood lymphocyte subsets in treatment-naïve thalassemia patients from Wenshan Zhuang Zu and Miao Zu Autonomous Prefecture, Yunnan Province, China (geographic coordinates: 23.37°N, 104.25°E), with stratification by both genetic subtype and age group, while employing healthy controls for comparative assessment. This investigation aims to elucidate the intrinsic immunological profile of thalassemia patients in its unmodified state.

## Methods

### Patient recruitment

All 535 participants were from Wenshan Zhuang and Miao Autonomous Prefecture, Yunnan Province, China (104.25°E, 23.37°N). Inclusion criteria: Genetically confirmed thalassemia patients or healthy controls with normal genotypes; Treatment-naïve (without prior blood transfusion, splenectomy, iron chelation therapy, or hematopoietic stem cell transplantation); Complete clinical data and valid informed consent obtained from participants or their legal guardians. Exclusion criteria: Participants with diabetes, hypertension, hyperlipidemia, tumors, autoimmune diseases, acute or chronic infections, trauma, or other genetic diseases were excluded. Venous blood samples (8–10 mL) were collected from each patient into ethylenediaminetetraacetic acid (EDTA) anticoagulant tubes. All specimens were transported to the testing center within 8–10 h under cold-chain conditions at 2-8 °C. Aliquots of 4–5 mL were separately allocated for lymphocyte subset analysis and complete blood count, while another 4–5 mL was used for NGS assay. All the patients’ tests were conducted at The First People’s Hospital of Yunnan Province.

### Genetic testing

In brief, venous blood samples from subjects were collected in tubes containing EDTA. Genomic DNA was subsequently extracted from peripheral venous blood. As previously reported, all patients underwent screening for α- and β-globin gene mutations through NGS (10).

### Lymphocyte subsets analysis

Flow cytometry was employed to quantitatively assess lymphocyte subsets in peripheral blood samples from the study population. Total T cells (CD3+ T cells) were identified as CD45+CD3+; CD4+ T cells as CD45+CD3+CD4+CD8-; CD8+ T cells as CD45+CD3+CD4-CD8+; B cells as CD45+CD3-CD19+; NK cells as CD45+CD3-CD16+CD56+; and regulatory T cells(Tregs) as CD45+CD3+CD4+CD25+CD127dim. All antibody reagents were sourced from Agilent, including: CD3/CD16+CD56/CD45/CD19 detection kit (Flow cytometry-FITC/PE/PerCP/APC, clone numbers: SK7/3G8+HCD56/HI30/HIB19); CD3/CD8/CD45/CD4 detection kit (Flow cytometry-FITC/PE/PerCP/APC, clone numbers: SK7/SK1/HI30/SK3); and CD127 detection reagent (Flow cytometry-PE, clone number: A019D5).

### Statistical analysis

The data were first subjected to normality testing. For normally distributed data, independent samples t-tests were employed, while non-parametric tests were used for non-normally distributed data (denoted by “#”). Age was described using quartiles and mean ± standard deviation, and all lymphocyte data were presented as mean ± standard deviation, with values rounded to two decimal places. IBM SPSS Statistics 21.0 was utilized for differential analysis, and all graphs were generated using GraphPad Prism 9.5.1. p < 0.05 indicated statistical differences. The sample size included in the analysis was all equal to or greater than 5.

## Results

### Basic patient information

A total of 535 subjects were enrolled in this study. NGS testing revealed that 307 individuals were healthy controls, including 104 pediatric (<18 years) and 203 adults (≥18 years). Among the remaining 228 thalassemia patients, 123 had α-thalassemia (63 pediatric, 60 adult), 83 had β-thalassemia (53 pediatric, 30 adult), and 22 hadα+β-thalassemia (12 pediatric,10 adult). The mean corpuscular volume (MCV), mean corpuscular hemoglobin (MCH), hemoglobin (HGB) levels, and age ranges for all participants are presented in **Supplementary Table 1**. Among the α-thalassemia patients: 37 were α-silent carriers (7 with αα/-α4.2, 23 with αα/-α3.7, and 7 heterozygous for Hb Westmead). 77 were α-trait carriers (56 with αα/--SEA, 16 heterozygous for Hb Constant Spring, 3 with -α3.7/-α3.7, 1 heterozygous for Hb Quong Sze, and 1 with αCSα/-α4.2). 9 had α-Hb H disease (7 with -α3.7/--SEA, 1 with -α4.2/--SEA, and 1 with αCSα/--SEA). Among the β-thalassemia patients: 67 had β0-thalassemia (24 heterozygous for Codons 41/42 (-TTCT) β0, 42 heterozygous for Codon 17 (A>T) β0, and 1 heterozygous for IVS-I-1 (G>T) β0). 16 had β+-thalassemia (3 heterozygous for IVS-II-654 (C>T) β+, 2 heterozygous for Hb E β+, 5 heterozygous for Codons 71/72 (+A) β0, 4 heterozygous for -28 (A>G) β+, and 2 heterozygous for -50 (G>A) β+). Among the combined α- and β-thalassemia patients: 4 were heterozygous for αα/--SEA and Codon 17 (A>T) β0. 4 were heterozygous for αα/-α3.7 and Codons 41/42 (-TTCT) β0. 2 were heterozygous for αα/-α3.7 and Codon 17 (A>T) β0. 2 were heterozygous for αα/-α3.7 and IVS-I-1 (G>T) β0. 2 were heterozygous for αα/--SEA and -28 (A>G) β+. 2 were heterozygous for αα/-α4.2 and Codons 41/42 (-TTCT) β0. 1 was heterozygous for Hb Westmead and Codons 41/42 (-TTCT) β0. 1 was heterozygous for Hb Constant Spring and IVS-I-1 (G>T) β0. 1 was heterozygous for -α3.7/--SEA and Codon 17 (A>T) β0. 1 was heterozygous for αα/-α3.7 and -50 (G>A) β+. 1 was heterozygous for αα/-α3.7 and Hb E β+. 1 was compound heterozygous for αα/-α3.7 and Codon 17 (A>T) β0, as well as Hb E β+. Additional detailed patient information is provided in **Table 1**, **Supplementary Table 1**.

**Table 1:** Basic patient information.

Patient type		All (n=535)	Gender (male/female)	Pediatric	Gender (male/female)	Adult	Gender (male/female)
	Normal population	307	91/216	104	54/50	203	37/166
α-thalassemia	α-silent	37	15/24	17	8/9	20	7/13
	αα/-α4.2	7	2/5	4	1/3	3	1/2
	αα/-α3.7	23	11/12	13	7/6	10	4/6
	Heterozygous for Hb Westmead	7	2/5	—		7	2/5
	α-Trait	77	24/53	37	18/19	40	6/34
	αα/--SEA	56	19/37	30	14/16	26	5/21
	-α3.7/-α3.7	3	1/2	1	1/0	2	0/2
	Heterozygous for Hb Quong Sze	1	0/1	—		1	0/1
	Heterozygous for Hb Constant Spring	16	4/12	5	3/2	11	1/10
	αCSα/-α4.2	1	0/1	1	0/1	—	—
	α-Hb H disease	9	6/3	9	6/3	—	—
	-α3.7/--SEA	7	4/3	7	4/3	—	—
	-α4.2/--SEA	1	1/0	1	1/0	—	—
	α CSα/--SEA	1	1/0	1	1/0	—	—
β-thalassemia	β0	67	28/39	47	27/20	20	1/19
	Heterozygous for Codons 41/42 (-TTCT) β0	24	12/12	19	11/8	5	1/4
	Heterozygous for Codon 17 (A>T) β0	42	16/26	28	16/12	14	0/14
	Heterozygous for IVS-I-1 (G>T) β0	1	0/1	—		1	0/1
	β+	16	3/12	6	3/3	10	0/10
	Heterozygous for IVS-II-654 (C>T) β+	3	0/3	—	—	3	0/3
	Heterozygous for Hb E β+	2	0/2	—	—	2	0/2
	Heterozygous for Codons 71/72 (+A) β0	5	1/4	1	1/0	4	0/4
	Heterozygous for -28 (A>G) β+	4	2/2	3	2/1	1	0/1
	Heterozygous for -50 (G>A) β+	2	0/2	2	0/2	—	—
α+β-thalassemia	α+β-thalassemia	22	6/16	12	6/6	10	0/10
	Heterozygous for αα/-α3.7 and Codon 17 (A>T) β0, Heterozygous for Hb E β+	1	0/1	1	0/1	—	—
	αα/-α3.7 and Heterozygous for Hb E β+	1	1/0	1	1/0	—	—
	αα/-α3.7and Heterozygous for -50 (G>A) β+	1	1/0	1	1/0	—	—
	- α3.7/--SEA and Heterozygous for Codon 17 (A>T) β0	1	0/1	1	0/1	—	—
	Heterozygous for Hb Constant Spring and Heterozygous for IVS-I-1 (G>T) β0	1	0/1	1	0/1	—	—
	αα/-α4.2 and Heterozygous for Codons 41/42 (-TTCT) β0	2	0/2	—	—	2	0/2
	Heterozygous for Hb Westmead and Heterozygous for Codons 41/42 (-TTCT) β0	1	0/1	—	—	1	0/1
	αα/--SEA and Heterozygous for -28 (A>G) β+	2	0/2	—	—	2	0/2
	αα/-α3.7and Heterozygous for IVS-I-1 (G>T) β0	2	0/2	—	—	2	0/2
	αα/-α3.7 and Heterozygous for Codons 41/42 (-TTCT) β0	4	1/3	2	1/1	2	0/2
	αα/-α3.7 and Heterozygous for Codon 17 (A>T) β0	2	1/1	1	1/0	1	0/1
	αα/--SEA and Heterozygous for Codon 17 (A>T) β0	4	2/2	4	2/2	—	—

### Lymphocyte subsets in pediatric thalassemia patients

**Figure 1:**
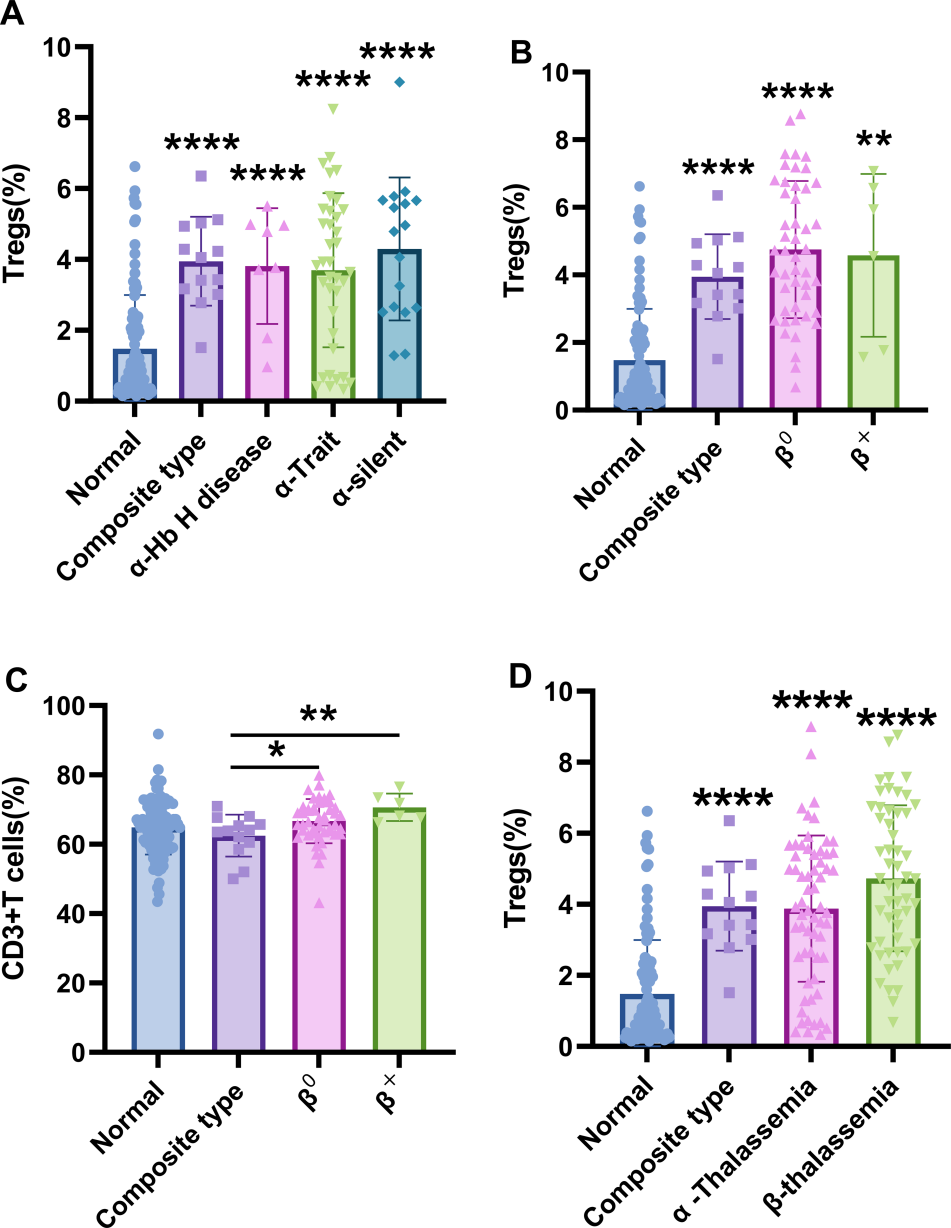
Differential expression of lymphocytes in pediatric populations. **(A, B)** Comparison of Tregs between α-thalassemia patients **(A)** and β-thalassemia patients **(B)** versus Normal controls. C: Expression of CD3+ T cells in β-thalassemia patients. **(D)** Comparison of Tregs between thalassemia patients and Normal controls. *P<0.05, **P<0.01, ****P<0.0001.

Our analysis of lymphocytes in pediatric patients with α-thalassemia revealed that the frequency of Tregs was significantly higher in the α-silent (4.30 ± 2.02%), α-Trait (3.70 ± 2.18%), α-Hb H disease (3.81 ± 1.63%), and α+β-thalassemia (3.95 ± 1.35%) groups compared to healthy controls (1.48 ± 1.52%) (P < 0.0001; **Table 2**; **Figure 1A**). No statistically significant differences were observed in CD3+ T cells, CD4+ T cells, CD8+ T cells, B cells, or NK cells among the other groups (P > 0.05) (**Table 2**). Further analysis of lymphocytes in pediatric patients β-thalassemia showed that Tregs proportions were significantly elevated in β0 (4.75 ± 2.03%) and β+ (4.58 ± 2.41%) patients compared to healthy pediatric (1.48 ± 1.52%) (P < 0.0001) (**Table 2**) (**Figure 1B**). Additionally, CD3+ T cell percentages in α+β-thalassemia patients (62.50 ± 6.03%) were significantly lower than in β0 (66.69 ± 6.38%) and β+ (70.66 ± 3.95%) patients (**Table 2**) (**Figure 1C**). No significant differences were found in CD3+ T cells, CD4+ T cells, CD8+ T cells, B cells, or NK cells among other groups (P > 0.05) (**Table 2**).

**Table 2:** Comparison of lymphocyte subset distributions among pediatric patients with thalassemia.

Patient type		CD3+ T cells	CD4+ T cells	CD8+ T cells	B cells	NK cells	Tregs
Normal (Healthy pediatric) (%)		64.88 ± 7.81 (%)	36.33 ± 7.59 (%)	22.16 ± 6.57 (%)	14.63 ± 7.43 (%)	15.95 ± 7.39 (%)	1.48 ± 1.52 (%)
α+β-thalassemia		62.50 ± 6.03 (%)	35.60 ± 7.17 (%)	21.06 ± 3.29 (%)	15.38 ± 6.03 (%)	15.46 ± 8.22 (%)	3.95 ± 1.35 (%)
α-thalassemia	α-silent	65.99 ± 8.47 (%)	37.79 ± 6.40 (%)	20.83 ± 7.14 (%)	13.61 ± 7.43 (%)	15.51 ± 8.81 (%)	4.30 ± 2.02 (%)
	α-Trait	63.26 ± 8.95 (%)	35.88 ± 6.30 (%)	22.10 ± 6.36 (%)	14.49 ± 6.68 (%)	16.26 ± 7.90 (%)	3.70 ± 2.18 (%)
	α-Hb H disease	67.09 ± 8.90 (%)	39.06 ± 12.92 (%)	22.59 ± 7.20 (%)	12.19 ± 4.62 (%)	14.79 ± 6.85 (%)	3.81 ± 1.63 (%)
	P (α-Trait VS α-Hb H disease)	0.279	0.517	0.848	0.359	0.627	0.891#
	P (α-Trait VSα+β-thalassemia)	0.776	0.897	0.579	0.676	0.756	0.698#
	P (α-Trait VS Normal population	0.301	0.745	0.963	0.923	0.826	<0.0001#
	α-Hb H disease VS α+β-thalassemia	0.173	0.504	0.587	0.215	0.849	0.830
	α-Hb H disease VS Normal population	0.446	0.573	0.859	0.362	0.669	<0.0001#
	α+β-thalassemia VS Normal population	0.292	0.744	0.337	0.727	0.825	<0.0001#
	P(α-silent VS α-Trait)	0.295	0.306	0.516	0.664	0.756	0.341#
	P(α-silent VS α-Hb H disease)	0.768	0.744	0.573	0.6250	0.8390	0.558
	P(α-silent VSα+β-thalassemia)	0.218	0.386	0.907	0.489	0.986	0.589
	P(α-silent VS Normal population)	0.592	0.454	0.448	0.600	0.828	<0.0001#
β-thalassemia	β0	66.69 ± 6.38 (%)	34.27 ± 8.73 (%)	23.52 ± 6.21 (%)	14.73 ± 6.80 (%)	14.57 ± 6.15 (%)	4.75 ± 2.03 (%)
	β+	70.66 ± 3.95 (%)	37.66 ± 9.15 (%)	25.83 ± 8.26 (%)	13.45 ± 9.49 (%)	10.78 ± 6.33 (%)	4.58 ± 2.41 (%)
	P(β0 VS β+)	0.146	0.377	0.413	0.678#	0.162#	0.847
	P(β0 VS α+β-thalassemia)	0.038	0.616	0.175	0.756	0.672	0.088
	P(β0 VS Normal population)	0.164	0.143	0.230	0.937	0.269	<0.0001#
	P(β+ VS α+β-thalassemia)	0.008	0.601	0.224	0.594#	0.236#	0.566
	P(β+ VS Normal population )	0.076	0.681	0.192	0.709#	0.097#	<0.0001#

To validate this finding across different genotypes, we analyzed the pediatric cohort. We confirmed that Treg frequencies were consistently higher in all thalassemia subtypes (α-, β-, and α+β-thalassemia) compared to healthy controls (**Figure 1D**).

### Lymphocyte subsets in adult thalassemia patients

**Figure 2:**
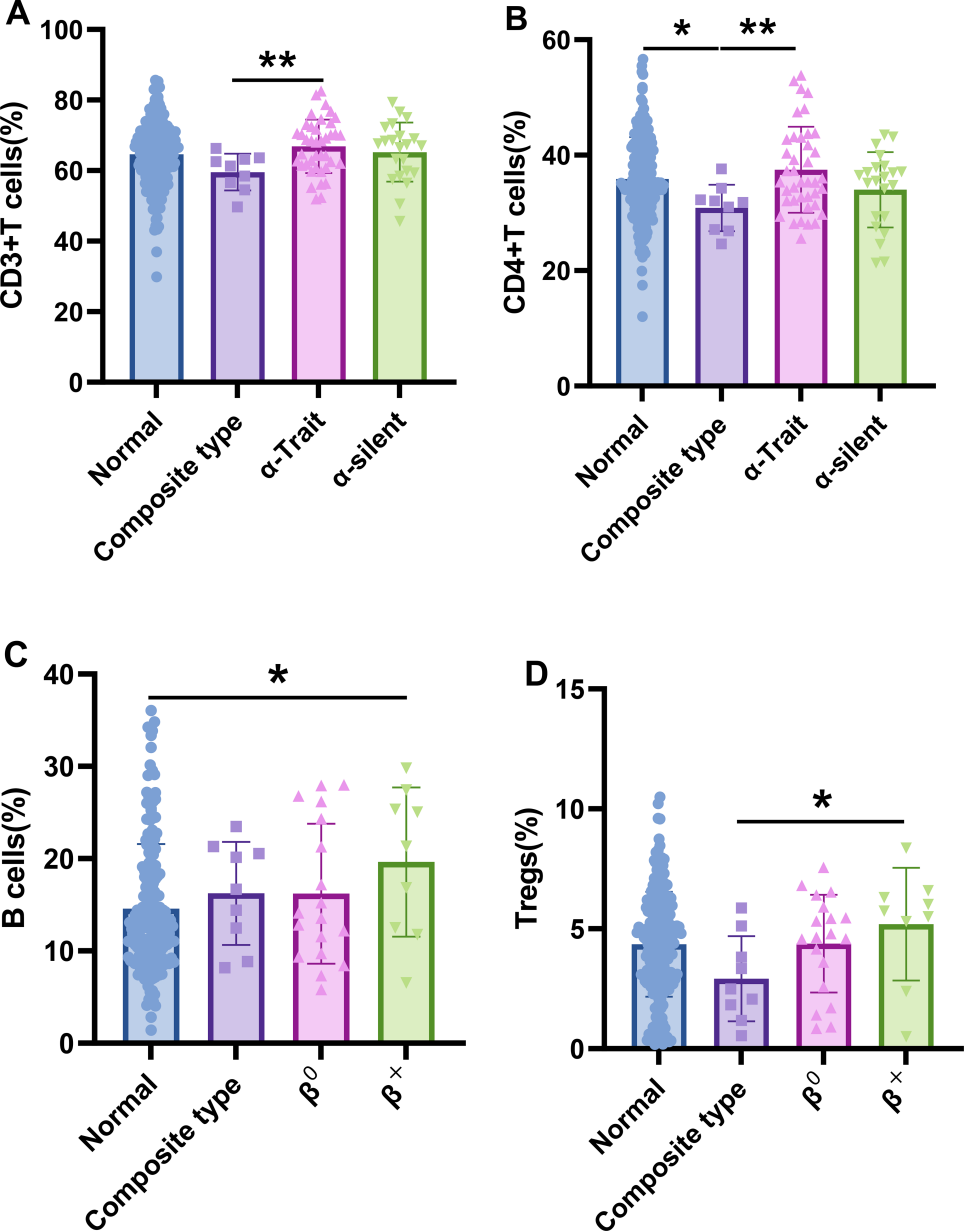
Differences in lymphocyte subsets among adult populations. **(A)** Differences in CD3+ T cells between compound type and α-Trait; **(B)** Differences in CD4+ T cells among normal adults, compound type, and α-Trait; **(C)** Differences in B cells between normal adults and β+; **(D)** Differences in T-regs between compound type and β+. *P<0.05, **P<0.01.

In our cohort, we observed that adult α-thalassemia patients with α+β-thalassemia exhibited significantly lower proportions of CD3+ T cells (59.58 ± 5.24%) and CD4+ T cells (30.85 ± 4.0%) compared to adult α-Trait carriers (P = 0.009, P = 0.001) (**Table 3**) (**Figures 2A, B**). In contrast, healthy adults demonstrated a significantly higher proportion of CD4+ T cells (35.88 ± 7.23%) than α+β-thalassemia patients (30.85 ± 4.0%) (P = 0.040) (**Figure 2B**). No statistically significant differences were observed among other groups for CD3+ T cells, CD4+ T cells, CD8+ T cells, T-regs, B cells, or NK cells (P>0.05) (**Table 3**).

**Table 3:** Differences among various types of thalassemia in the adult group.

Patient type		CD3+ T cells	CD4+ T cells	CD8+ T cells	B cells	NK cells	Tregs
Normal (Healthy adult)		64.61 ± 9.19(%)	35.88 ± 7.23(%)	22.76 ± 7.41(%)	14.58 ± 7.01(%)	16.22 ± 8.66(%)	4.36 ± 2.19(%)
α+β-thalassemia		59.58 ± 5.24(%)	30.85 ± 4.0(%)	22.56 ± 3.08(%)	16.24 ± 5.58(%)	18.51 ± 5.69(%)	2.92 ± 1.78(%)
α-thalassemia	α-silent	65.25 ± 8.37(%)	34.01 ± 6.53(%)	24.13 ± 6.62(%)	13.72 ± 8.68(%)	14.86 ± 7.03(%)	4.12 ± 2.54(%)
	α-Trait	66.86 ± 7.58(%)	37.48 ± 7.45(%)	23.34 ± 7.32(%)	12.63 ± 6.84(%)	15.93 ± 7.42(%)	4.26 ± 2.00(%)
	P(α-silent VS α-Trait)	0.443	0.072	0.675	0.588#	0.583	0.802#
	P(α-silent VS α+β-thalassemia)	0.071	0.190	0.503	0.430#	0.179	0.208#
	P(α-silent VS Normal)	0.757	0.245	0.407	0.592#	0.478#	0.632#
	P(α-Trait VS α+β-thalassemia)	0.009	0.001	0.756	0.148#	0.334	0.079
	P (α-Trait VS Normal)	0.148	0.206	0.652	0.107#	0.841#	0.805
	P (α+β-thalassemia VS Normal)	0.105	0.040	0.862	0.487	0.435#	0.054
β-thalassemia	β0	63.30 ± 9.97(%)	32.10 ± 10.88(%)	20.04 ± 8.52(%)	15.37 ± 8.21(%)	14.72 ± 9.39(%)	4.38 ± 2.04(%)
	β+	61.86 ± 6.19(%)	35.87 ± 7.93(%)	20.01 ± 5.76(%)	19.63 ± 8.07(%)	14.63 ± 6.90(%)	5.19 ± 2.35(%)
	β0 VS β+	0.695	0.364	0.991	0.209	0.979#	0.354
	P (β0 VS α+β-thalassemia)	0.305	0.743	0.401	0.779	0.277#	0.077
	P (β0 VS Normal)	0.556	0.265	0.132	0.644	0.473#	0.967
	P (β+ VS α+β-thalassemia)	0.412	0.111	0.258	0.314	0.212	0.034
	P (β+ VS Normal)	0.375	0.994	0.272	0.037	0.586#	0.264

Subsequently, we analyzed lymphocyte subsets in adult β-thalassemia patients. Notably, β+ adult patients with thalassemia displayed a significantly higher proportion of B cells (19.63 ± 8.07%) compared to healthy adults (14.58 ± 7.01%) (P = 0.037) (**Table 3**) (**Figure 2C**). Additionally, the proportion of T-regs was significantly higher in β+ adult patients with thalassemia (5.19 ± 2.35%) than in α+β-thalassemia patients (2.92 ± 1.78%) (P = 0.034) (**Table 3**) (**Figure 2D**). Apart from these alterations, no other significant intergroup differences were detected for CD3+ T cells, CD4+ T cells, CD8+ T cells, or NK cells (P>0.05) (**Table 3**). These findings suggest that while most lymphocyte subsets remain relatively stable in adult thalassemia patients, there are specific alterations in B cells and T-regs.

### Differences between pediatric and adult patients

**Figure 3:**
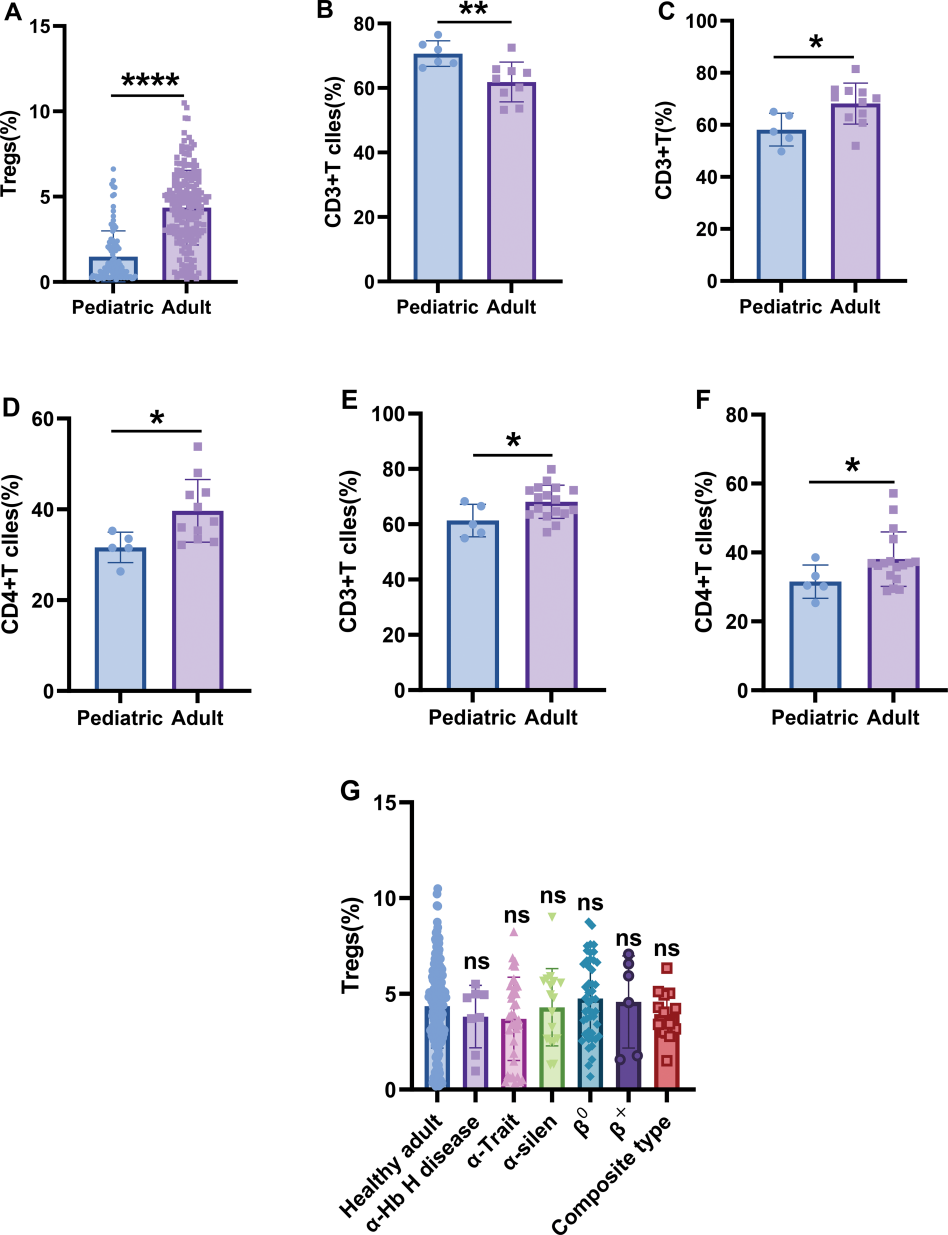
Comparison between pediatric patients and adults. **(A)** Tregs levels in healthy adults versus healthy children. **(B)** CD3+ T cell levels in pediatric versus adult β+patients. **(C, D)** CD3+ **(C)** and CD4+ **(D)** T cell levels in pediatric versus adult patients heterozygous for Hb Constant Spring. **(E, F)** CD3+ **(E)** and CD4+ **(F)** T cell levels in pediatric versus adult patients heterozygous for the Codons 41/42 (-TTCT) β0 mutation Codons 41/42 (-TTCT) β0 mutation. **(G)** Expression of Tregs in different patient types. ns, No statistical difference *P<0.05, **P<0.01, ****P<0.0001.

To further investigate potential differences in these lymphocytes between adults and pediatric patients, we conducted a comparative analysis of the same lymphocyte subtypes in these two patient groups. Due to insufficient sample sizes for certain mutations(n<5) that did not meet statistical requirements (**Table 1**), we focused our analysis on αα/-α4.2, αα/-α3.7, αα/--SEA, Hb Constant Spring heterozygotes, Codons 41/42 (-TTCT) β0 heterozygotes, and Codon 17 (A>T) β0 heterozygotes. In healthy individuals, Treg proportions were significantly higher in adults (4.36 ± 2.19%) compared to pediatric controls (1.48 ± 1.52%) (P < 0.0001; **Supplementary Table 2**; **Figure 3A**). Among β+ patients, CD3+ T cells were lower in adults (35.87 ± 7.93%) than in pediatric patients (37.66 ± 9.15%) (P = 0.009) (**Supplementary Table 2**) (**Figure 3B**). No statistically significant differences were observed for other lymphocyte subtypes (P > 0.05) (**Supplementary Table 2**).

Subgroup analysis by genotype revealed that in Hb Constant Spring heterozygotes, adult patients exhibited significantly higher proportions of CD3+ T cells (68.19 ± 7.86%) and CD4+ T cells (39.67 ± 6.92%) compared to pediatric patients (CD3+ T cells: 58.14 ± 6.28%, CD4+ T cells: 31.62 ± 3.36%) (P = 0.025, P = 0.029) (**Supplementary Table 3**) (**Figures 3C, D**). Conversely, in Codons 41/42 (-TTCT) β0 heterozygotes, pediatric patients demonstrated higher proportions of CD3+ T cells (68.13 ± 6.02%) and CD4+ T cells (38.08 ± 7.88%) than adult patients (CD3+ T cells: 61.35 ± 5.86%, CD4+ T cells: 31.54 ± 4.81) (P = 0.038, P = 0.044) (**Supplementary Table 3**) (**Figures 3E, F**). No significant differences were observed in other mutation types (**Supplementary Table 3**).

As illustrated in **Figure 1D**, Tregs were elevated in pediatric patients, and healthy adults also showed higher Tregs than healthy pediatric (**Figure 3A**). To determine whether Tregs in pediatric patients differed from those in healthy adults, we conducted further comparisons and found no significant difference. This suggests that elevated Tregs in thalassemia-afflicted pediatric patients represent a common feature, potentially indicative of a pathological process underlying disease progression.

## Discussion

Our study comprehensively characterized the distribution of lymphocyte subsets in untreated adult and pediatric patients with thalassemia, including various genotypes and age groups, in comparison with healthy individuals. We found that Tregs were significantly lower in healthy pediatric individuals than in healthy adults. Tregs were significantly elevated in pediatric thalassemia patients compared with healthy pediatric individuals, while no significant difference was observed between pediatric thalassemia patients and healthy adults. In addition, CD3+ T cells were lower in pediatric patients with compound thalassemia than in those with β-thalassemia. Pediatric β+ patients exhibited lower CD3+ T cells than adult β+ patients. Pediatric patients with Hb Constant Spring heterozygous mutations and Codons 41/42 (-TTCT) β0 heterozygous mutations also showed lower CD3+ T and CD4+ T cells than adult patients. Furthermore, B cells were higher in adult β+ patients than in healthy adults, and CD4+ T cells were lower in compound patients than in healthy controls.

Current research on thalassemia primarily focuses on splenectomy treatment for patients with severe β-thalassemia. Studies indicate that the percentages of B cells and T cells in adult patients post-splenectomy are significantly higher than in healthy individuals (4–7). Additionally, β-thalassemia patients exhibit markedly increased proportions of CD8+CD28- and CD3+CD95+ T lymphocytes (11), while the reduction in IL-2-expressing T cells may reflect a decline in naive and central memory T cells (12). In pediatric patients who have not undergone splenectomy, the proportion of apoptotic T lymphocytes is elevated (13). These findings also indicate that thalassemia patients have abnormal immune cell characteristics.

As negative regulatory cells of the immune system, Tregs play a central role by maintaining immune tolerance and homeostasis, with their main functions including suppression of excessive inflammatory responses and autoimmune damage (14–17). Dysfunction of Tregs can impair anti-infection and anti-tumor capabilities, exerting negative effects on immune function (18, 19). Previous studies have shown that increased Tregs numbers were observed in pediatric patients with severe β-thalassemia who were chronically exposed to antigen stimulation due to repeated blood transfusions (20, 21). However, further research has revealed that elevated Tregs levels are not limited to repeatedly transfused patients, as this phenomenon also occurs in transfusion-naïve patients with severe β-thalassemia (6, 7, 22, 23). In pediatric patients with transfusion-dependent thalassemia major (TDT), increased Tregs may further alter immune status and suppress active immune function (7, 22, 23). Studies that did not stratify pediatric and adult groups also found elevated Treg percentages and absolute counts in TDT patients, showing positive correlation with serum ferritin levels (24). Notably, although the quantity of Tregs is increased in pediatric patients with severe β-thalassemia, their suppressive function is significantly weaker compared to Tregs from healthy individuals (25).

Furthermore, we observed that Tregs levels in healthy pediatric individuals were significantly lower than those in healthy adults (**Supplementary Table 2**; **Figure 3A**). Studies have demonstrated that with advancing age, the number of newly generated tTregs and recirculating pTregs increases in murine thymic Tregs (26). Additionally, thymic involution in aged mice promotes Tregs differentiation (27). Prior to this study, large-scale human validation data were lacking, and our research provides direct evidence that adult Treg levels are higher than those in pediatric individuals (**Supplementary Table 2**; **Figure 3A**). Notably, Treg levels in pediatric thalassemia patients were not only higher than those in healthy pediatric individuals but also comparable to healthy adults.

In summary, our study systematically characterized CD3+ T, CD4+ T, CD8+ T, B, NK, and Tregs in untreated thalassemia patients. We discovered that elevated Tregs in pediatric thalassemia patients represent a common phenomenon, suggesting altered immune profiles in these young patients. Furthermore, our findings supplement the understanding that Tregs expression is age-dependent. These results may provide important references for comprehending the immune status of thalassemia patients and for further clinical management and research.

### Study limitations

Despite providing a comprehensive analysis of lymphocyte subsets across different age groups and thalassemia genotypes, our study is subject to several limitations. First, the cross-sectional design precludes causal inferences regarding the relationship between immune profiles and clinical outcomes. Additionally, the lack of detailed longitudinal clinical data (such as infection history, iron overload markers, and clinical endpoints) limits the scope of our analysis. Second, the sample size in certain subgroups, particularly for specific rare genotypes, was relatively small. This may introduce statistical bias and prevented us from conducting more in-depth stratified analyses. Finally, as our data were derived exclusively from a single region (Wenshan, Yunnan), the generalizability of our findings to other populations may be limited.

## Data Availability

The original contributions presented in the study are included in the article/**Supplementary Material**. Further inquiries can be directed to the corresponding authors.
