# Supplementary material for: Lymphocyte subsets in untreated thalassemia patients: differences by genotype and age
Source: Front Immunol. 2026 Jun 3;17:1832294. doi: 10.3389/fimmu.2026.1832294 (PMC13272325; doi:10.3389/fimmu.2026.1832294)
Supplement: Supplementary file 1 [file Table1.docx]

Supplement Table 1. Data on MCV, MCH, HGB and age of the population.

|  |  | MCV (fL) | MCH (pg) | HGB(g/L) | Age (mean ± standard deviation) | Age (interquartile) |
| --- | --- | --- | --- | --- | --- | --- |
| α-silent | Pediatric | 81.62±7.85 | 23.36±3.10 | 116.53±15.42 | 3.99±6.14 | 1.00(0.65-3.50) |
|  | Adult | 93.63±10.37 | 26.93±2.65 | 131.27±18.08 | 25.86±4.88 | 26.00(21.75-29.25) |
| α-Trait | Pediatric | 67.44±14.18 | 19.71±4.82 | 110.10±21.39 | 2.65±3.68 | 1.00(0.70-3.00) |
|  | Adult | 80.54±12.00 | 23.54±2.61 | 123.69±15.88 | 27.25±5.32 | 26.00(24.00-30.50） |
| α-Hb H disease | Pediatric | 57.79±13.41 | 16.10±2.50 | 89.13±3.97 | 4.23±3.55 | 4.00(1.05-6.00) |
| β0 | Pediatric | 63.65±5.84 | 17.89±1.53 | 101.19±8.93 | 2.79±3.53 | 2.00(0.70-3.00) |
|  | Adult | 70.23±6.64 | 19.83±1.01 | 112.81±11.54 | 30.05±8.70 | 27.00(23.00-38.00) |
| β+ | Pediatric | 76.50±13.68 | 21.95±4.77 | 101.33±19.99 | 1.47±1.85 | 0.60(0.40-2.75) |
|  | Adult | 81.45±18.07 | 23.90±5.64 | 118.88±20.34 | 27.33±4.47 | 27.00(22.50-31.50) |
| α+β-thalassemia | Pediatric | 62.88±9.09 | 18.83±2.87 | 107.75±16.76 | 4.29±5.16 | 2.00(0.80-6.50) |
|  | Adult | 77.50±8.98 | 22.18±1.66 | 123.25±14.30 | 26.89±3.79 | 26.00(24.00-30.50) |
| Normal population | Pediatric | 82.21±13.67 | 23.67±4.90 | 110.23±20.69 | 3.18±4.56 | 1.00（0.70-3.00） |
|  | Adult | 97.10±9.50 | 29.65±3.14 | 132.38±22.31 | 26.25±6.05 | 25.00(22.00-30.00) |

Supplement Table 2. P-values between pediatric patients and adults

|  |  | CD3+ T cells | CD4+ T cells | CD8+ T cells | B cells | NK cells | Tregs |
| --- | --- | --- | --- | --- | --- | --- | --- |
| Normal population | Pediatric | 64.88±7.81 | 36.33±7.59 | 22.16±6.57 | 14.63±7.43 | 15.95±7.39 | 1.48±1.52 |
|  | Adult | 64.61±9.19 | 35.88±7.23 | 22.76±7.41 | 14.58±7.01 | 16.22±8.66 | 4.36±2.19 |
|  | P | 0.802 | 0.615 | 0.480 | 0.958 | 0.781# | ＜0.0001# |
| α-silent | Pediatric | 65.99±8.47 | 37.79±6.40 | 20.83±7.14 | 13.61±7.43 | 15.51±8.81 | 4.30±2.02 |
|  | Adult | 65.25±8.37 | 34.01±6.53 | 24.13±6.62 | 13.72±8.68 | 14.86±7.03 | 4.12±2.54 |
|  | P | 0.786 | 0.079 | 0.144 | 0.966# | 0.799# | 0.813# |
| α-Trait | Pediatric | 63.26±8.95 | 35.88±6.30 | 22.10±6.36 | 14.49±6.68 | 16.26±7.90 | 3.70±2.18 |
|  | Adult | 66.86±7.58 | 37.48±7.45 | 23.34±7.32 | 12.63±6.84 | 15.93±7.42 | 4.26±2.00 |
|  | P | 0.060 | 0.313 | 0.430 | 0.231# | 0.849# | 0.238# |
| β0 | Pediatric | 66.69±6.38 | 34.27±8.73 | 23.52±6.21 | 14.73±6.80 | 14.57±6.15 | 4.75±2.03 |
|  | Adult | 63.30±9.97 | 32.10±10.88 | 20.04±8.52 | 15.37±8.21 | 14.72±9.39 | 4.38±2.04 |
|  | P | 0.104 | 0.399 | 0.069 | 0.744# | 0.950# | 0.501 |
| β+ | Pediatric | 70.66±3.95 | 37.66±9.15 | 25.83±8.26 | 13.45±9.49 | 10.78±6.33 | 4.58±2.41 |
|  | Adult | 61.86±6.19 | 35.87±7.93 | 20.01±5.76 | 19.63±8.07 | 14.63±6.90 | 5.19±2.35 |
|  | P | 0.009 | 0.693 | 0.130 | 0.197# | 0.295# | 0.630 |
| α+β-thalassemia | Pediatric | 62.50±6.03 | 35.60±7.17 | 21.06±3.29 | 15.38±6.03 | 15.46±8.22 | 3.95±1.35 |
|  | Adult | 59.58±5.24 | 30.85±4.0 | 22.56±3.08 | 16.24±5.58 | 18.51±5.69 | 2.92±1.78 |
|  | P | 0.254 | 0.088 | 0.295 | 0.740 | 0.348# | 0.126# |

Supplement Table 3. Comparison of different genes between adults and pediatric

|  |  | CD3+ T cells (%) | CD4+ T cells (%) | CD8+ T cells (%) | B cells (%) | NK cells (%) | Tregs (%) |
| --- | --- | --- | --- | --- | --- | --- | --- |
| αα/-α4.2 | Pediatric | 68.37±5.49 | 40.03±5.90 | 22.73±8.28 | 7.02±2.85 | 16.16±4.24 | 4.01±1.70 |
|  | Adult | 67.09±0.55 | 35.73±5.60 | 21.48±3.00 | 10.57±5.84 | 13.82±2.65 | 4.67±3.31 |
|  | P | 0.673 | 0.376 | 0.817 | 0.329 | 0.443 | 0.739# |
| αα/-α3.7 | Pediatric | 65.25±9.25 | 37.10±6.61 | 20.25±7.01 | 15.64±7.25 | 15.31±9.94 | 4.39±2.16 |
|  | Adult | 64.07±7.92 | 33.25±7.59 | 24.80±5.11 | 14.90±8.6 | 14.35±7.45 | 3.84±2.30 |
|  | P | 0.748 | 0.208 | 0.099 | 0.827# | 0.800# | 0.562# |
| αα/--SEA | Pediatric | 63.79±9.35 | 36.06±6.30 | 22.57±6.87 | 15.07±6.95 | 15.47±8.26 | 3.53±2.33 |
|  | Adult | 66.72±7.26 | 36.22±7.82 | 24.27±7.51 | 13.05±6.94 | 15.78±6.88 | 4.62±1.72 |
|  | P | 0.203 | 0.929 | 0.381 | 0.283 | 0.877# | 0.055# |
| Hb Constant Spring heterozygous | Pediatric | 58.14±6.28 | 31.62±3.36 | 20.92±2.84 | 12.03±5.92 | 21.54±4.72 | 3.93±1.09 |
|  | Adult | 68.19±7.86 | 39.67±6.92 | 22.34±7.16 | 12.82±7.30 | 14.78±8.04 | 4.03±2.40 |
|  | P | 0.025 | 0.029 | 0.679 | 0.837# | 0.106# | 0.932# |
| Codons 41/42 (-TTCT) β0 heterozygous | Pediatric | 68.13±6.02 | 38.08±7.88 | 23.33±4.80 | 14.51±6.37 | 13.25±6.04 | 4.63±1.91 |
|  | Adult | 61.35±5.86 | 31.54±4.81 | 22.65±5.85 | 16.06±6.36 | 16.39±10.76 | 4.73±1.41 |
|  | P | 0.038 | 0.044 | 0.794 | 0.638 | 0.404# | 0.916 |
| Codon 17 (A>T) β0 heterozygous | Pediatric | 65.94±6.73 | 32.15±8.85 | 23.34±7.01 | 14.87±7.20 | 15.10±6.27 | 4.91±2.16 |
|  | Adult | 64.00±11.19 | 32.31±12.51 | 19.11±9.30 | 15.13±8.98 | 14.12±9.22 | 4.25±2.25 |
|  | P | 0.487 | 0.963 | 0.107 | 0.919# | 0.688# | 0.366 |
